# Prognostic Value of Lactate and Central Venous Oxygen Saturation after Early Resuscitation in Sepsis Patients

**DOI:** 10.1371/journal.pone.0153305

**Published:** 2016-04-07

**Authors:** Young Kun Lee, Sung Yeon Hwang, Tae Gun Shin, Ik Joon Jo, Gee Young Suh, Kyeongman Jeon

**Affiliations:** 1 Department of Critical Care Medicine, Samsung Medical Center, Sungkyunkwan University School of Medicine, Seoul, Republic of Korea; 2 Department of Emergency Medicine, Samsung Medical Center, Sungkyunkwan University School of Medicine, Seoul, Republic of Korea; 3 Division of Pulmonary and Critical Care Medicine, Department of Medicine, Samsung Medical Center, Sungkyunkwan University School of Medicine, Seoul, Korea; Hospital Sirio-Libanes, BRAZIL

## Abstract

The objective of this study was to evaluate the prognostic value of static and dynamic variables of central venous oxygen saturation (ScvO_2_) and lactate in patients with severe sepsis or septic shock who underwent early quantitative resuscitation. We also investigated whether ScvO_2_ measured after initial resuscitation could provide additive prognostic value to that of lactate. We analyzed the sepsis registry for patients presenting to the emergency department and included patients with simultaneous measurements of lactate and ScvO_2_ at the time of presentation (H0) and 6 hours (H6) after resuscitation. The primary outcome was 28-day mortality and multivariable logistic analysis was used to adjust for confounders. A total of 363 patients were included, and the overall 28-day mortality was 18%. The area under the receiver operator characteristic curve for predicting 28-day mortality was as follows: lactate (H6), 0.81; lactate (H0), 0.73; relative lactate change, 0.67; ScvO_2_ (H6), 0.65; relative ScvO_2_ change 0.59; ScvO_2_ (H0), 0.58. Patients with lactate normalization showed significantly lower 28-day mortality compared to patients without lactate normalization (3% vs. 28%, *P*<0.01). However, in those who achieved ScvO_2_ (H6) ≥70%, there was a significant difference in 28-mortality only in patients without lactate normalization (21% vs. 39%, *P<*0.01) but no difference in those with lactate normalization (4% vs. 3%, *P* = 0.71). In multivariable analysis, lactate normalization was significantly associated with 28-day mortality (adjusted odds ratio [OR] for 28-day mortality, 0.20; 95% confidence interval [CI], 0.07–0.54; *P* <0.01), but ScvO_2_ (H6) ≥70% showed only a marginal association (the adjusted OR for 28-day mortality, 0.51; 95% CI, 0.26–1.01; *P* = 0.05). ScvO_2_ (H6) ≥70% was associated with 28-day mortality only in cases without lactate normalization in subgroup analysis (adjusted OR 0.37, 95% CI, 0.18–0.79; *P* = 0.01). Six-hour lactate was the strongest predictor of 28-day mortality in patients with severe sepsis or septic shock. Six-hour ScvO_2_ provided additional prognostic value only in cases where lactate values were not normalized after resuscitation.

## Introduction

Despite international recommendations for early quantitative resuscitation in cases of severe sepsis or septic shock based on balancing systemic oxygen delivery with oxygen demand using targeted endpoints [1], the risk of death remains high [2, 3]. In addition, three recent large, multicenter, randomised clinical trials failed to demonstrate that early goal-directed therapy (EGDT) decreased mortality [4–6]. Therefore, there remains considerable debate regarding the relative value of early sepsis resuscitation goals, particularly the value of central venous oxygen saturation (ScvO_2_) [7]. However, there are limited data on the prognostic value of factors that could be used for guiding therapy after initial resuscitation [8].

Lactate has been studied for decades as a prognostic marker of resuscitation and patient outcome in sepsis [9]. Additionally, the prognostic value of lactate clearance (LC), or the decrease in lactate level in the first 6 hours after initial resuscitation with respect to death has been examined in severe sepsis and septic shock patients [10, 11]. However, there might be some pitfalls in the interpretation of LC and lactate concentrations because normal lactate levels are common even in patients with septic shock and lactate kinetics can be affected by many factors [12–16].

As static or dynamic indexes, ScvO_2_ and lactate levels might have prognostic value after initial resuscitation in patients with severe sepsis or septic shock, but discrepancies and misinterpretation of these results with regard to outcome prediction may also be common [8]. Therefore, we evaluated the prognostic value of static levels and changes in ScvO_2_ and lactate during the initial 6 hours after resuscitation in patients with severe sepsis or septic shock. We also investigated whether ScvO_2_ measured after initial resuscitation could be a useful predictor for outcome in addition to lactate in sepsis patients.

## Materials and Methods

This was a single-center, retrospective, observational cohort study of patients who presented to the emergency department (ED) at Samsung Medical Center (a 1,960-bed, university-affiliated, tertiary referral hospital in Seoul, South Korea) with severe sepsis or septic shock from August 2008 to March 2012. We analyzed the sepsis registry that was previously used in our studies of severe sepsis or septic shock [17–23]. The study was approved by the Institutional Review Board of Samsung Medical Center. The institutional review board waived the need for written informed consent from the patients because of the observational nature of the study. In addition, patient information was anonymized and de-identified prior to analysis.

### Study population

The study inclusion criteria were patients aged 18 years or older who presented with severe sepsis or septic shock. We excluded patients who met the following criteria: terminal malignancy, a previously signed “Do Not Resuscitate” (DNR) order, or refusal of EGDT. For the present study, the final study population included patients who underwent protocolized resuscitation with simultaneous measurement of serum lactate and ScvO_2_ at the time of presentation with severe sepsis or septic shock (H0) and 6 hours after resuscitation (H6).

### Definitions

Severe sepsis was defined as sepsis associated with acute organ dysfunction [24]. Septic shock was defined as sepsis with acute circulatory failure characterized by persistent hypotension (systolic arterial pressure <90 mmHg, mean arterial pressure (MAP) <60 mmHg, or a reduction in systolic blood pressure of more than 40 mmHg from baseline despite adequate volume resuscitation [24]. Relative ScvO_2_ change and relative lactate change were defined using the following formula: ([ScvO_2_ (H0)–ScvO_2_ (H6)]/ ScvO_2_ (H0)) x 100 (%) and ([lactate (H0)–lactate (H6)]/ lactate (H0)) x 100 (%), respectively. Lactate normalization was defined as a lactate concentration decrease to <2 mmol/L 6 hours after initial resuscitation or if both the initial and repeat lactate concentrations were <2 mmol/L.

According to the results of the 6-hour resuscitation period, including measurement of serum lactate and ScvO_2_ at H0 and H6, the study population was divided into four groups for comparison of outcomes. Patients with lactate normalization and ScvO_2_ ≥70% were assigned to group 1 and patients with lactate normalization and ScvO_2_ <70% were assigned to group 2. Patients without lactate normalization and ScvO_2_ ≥70% or ScvO_2_ <70% were assigned to groups 3 and 4, respectively.

### Resuscitation protocol and hemodynamic management

All patients were managed according to our EGDT protocol, which is an adaptation of the protocol outlined by Rivers et al. [25]. Once a patient met the criteria for severe sepsis or septic shock, fluid resuscitation and hemodynamic monitoring were initiated with placement of a central venous catheter with the internal jugular or subclavian vein approach for central venous pressure (CVP) and ScvO_2_ monitoring. Broad-spectrum antibiotics were administered as soon as possible. Hemodynamic resuscitation was conducted according to a prespecified treatment plan. First, isotonic crystalloid was administered in boluses to target CVP ≥8 mmHg. Second, if not achieved with fluid administration, systolic blood pressure ≥90 mmHg or MAP ≥65 mmHg, was targeted by initiating and titrating vasopressors (preferably norepinephrine as a first-line agent) to achieve this goal. Finally, ScvO_2_ ≥70% was targeted after CVP and blood pressure goals were met. If the ScvO_2_ was lower than 70% and hematocrit was lower than 30%, packed red blood cells were transfused to achieve a hematocrit of at least 30%. If the ScvO_2_ remained lower than 70% after the hematocrit reached 30% or higher, dobutamine was initiated and titrated in an attempt to achieve ScvO_2_ ≥70% at the treating physician’s discretion. Mechanical ventilation was initiated if necessary. However, subsequent lactate measurements after initiation of resuscitation were not used for goal-directed resuscitation. The resuscitation protocol was continued until all goals were achieved or for a maximum of 6 hours.

### Data collection

The following data were collected from our sepsis registry and electronic medical records: demographic data, infection source, type of shock, laboratory measurements including serial lactate and ScvO_2_ measurements, amount of fluid administered, and vasopressor dose. Sequential Organ Failure Assessment (SOFA) and Acute Physiology and Chronic Health evaluation (APACHE) II scores were evaluated to assess the severity of illness at the time of diagnosis of severe sepsis or septic shock from the obtained data. Finally, we documented the outcomes of septic patients including 28-day mortality, in-hospital mortality and length of hospital stay.

### Statistical analysis

All results are presented as the median and interquartile range (IQR) for numeric data and the number of patients (percentage) for categorical data. Comparisons for continuous variables were made using the Wilcoxon rank sum test or the Kruskal-Wallis test, and categorical data were tested using the chi-squared test. Multiple comparisons were performed to compare each group, and Bonferroni corrections were used to determine whether multiple comparisons were significant.

The receiver operator characteristic (ROC) curve with area under the ROC curve (AUC) was used to assess the usefulness of ScvO_2_ and lactate as static and dynamic variables to predict 28-day mortality.

Unadjusted and adjusted odds ratios (ORs) for primary outcome measures were calculated by univariable and multivariable logistic regression analysis, respectively. Variables that were found to be statistically significant at *P* <0.10 using univariable analysis were selected and included in the final multivariable models. The Hosmer-Lemeshow test was used to check the goodness of fit of the logistic regression. Kaplan-Meier curves were developed to assess the probability of survival according to lactate normalization and ScvO_2_ ≥70% achievement.

All tests were two-sided, and a *P* value less than 0.05 was considered significant. The data were analyzed using IBM SPSS 20 (IBM, Armonk, NY, USA) and STATA 13.0 (STATA Corporation, College Station, TX, USA).

## Result

During the study period, a total of 917 patients with severe sepsis or septic shock were entered into the sepsis registry. Of these, 147 patients were excluded according to the following exclusion criteria: terminal malignancy (n = 116), a signed DNR order (n = 27), or refusal of EGDT (n = 4). Finally, a total of 770 patients were eligible for this study. Of the eligible patients, 363 (47%) patients who met the inclusion criteria were included in the final analysis. Overall 28-day mortality was 18% in included patients, higher than that of excluded patients (13%, *P* = 0.08). The median APACHE II score of included patients was higher compared to excluded patients (18 vs. 16, respectively *P* <0.01)

Table 1 shows the baseline characteristics of the entire cohort and comparison between 28-day survivors and non-survivors. The initial SOFA and APACHE II scores were 8 (IQR, 6–10) and 17 (IQR, 13–22), respectively. The median ScvO_2_ (H0) and ScvO_2_ (H6) was 70% (IQR, 63–77) and 74% (IQR, 67–79), respectively. ScvO_2_ (H0) and ScvO_2_ (H6) were significantly higher in survivors. The median lactate (H0) and lactate (H6) concentration was 4.2 mmol/L (IQR, 2.4–5.7) and 2.4 mmol/L (IQR, 1.5–4.2), respectively. Lactate (H0) and lactate (H6) were significantly higher in non-survivors.

**Table 1:** Comparison of baseline characteristics of the study population (N = 363).

	Total (n = 363)	Survivor (n = 298)	Non-survivor (n = 65)	*P* value
Age, years	65 (54–72)	65 (54–72)	66 (55–75)	0.23
Gender, male	204 (56)	169 (57)	35 (54)	0.67
Comorbidities				
Hypertension	122 (34)	98 (33)	24 (37)	0.53
Diabetes	74 (20)	62 (21)	12 (19)	0.67
Chronic lung disease	20 (6)	18 (6)	2 (3)	0.34
Cardiovascular disease	32 (9)	24 (8)	8 (12)	0.27
Chronic renal failure	13 (4)	10 (3)	3 (5)	0.62
Liver cirrhosis	26 (46)	21 (7)	5 (8)	0.86
Malignancy	205 (57)	163 (55)	42 (65)	0.14
Site of infection				
Intra-abdomen	133 (37)	118 (40)	15 (23)	0.01
Respiratory tract	111 (31)	81 (27)	30 (46)	<0.01
Urinary tract	53 (15)	49 (16)	4 (6)	0.03
Others	66 (18)	50 (17)	16 (25)	0.14
Initial vital signs				
MAP (mmHg)	64 (56–73)	64 (56–73)	61 (52–74)	0.08
Heart rate (per minute)	113 (95–133)	112 (95–133)	115 (101–139)	0.17
Respiratory rate (per minute)	20 (20–24)	20 (20–22)	24 (20–30)	<0.01
Body temperature (°C)	38.0 (36.9–38.9)	38.2 (37–38.9)	37.2 (36.5–38.6)	<0.01
Laboratory tests				
Baseline lactate, mmol/L	4.2 (2.4–5.7)	3.9 (2.2–5.4)	5.7 (4.4–8.9)	<0.01
Six-hour lactate, mmol/L	2.4 (1.5–4.2)	2.1 (1.4–3.7)	5.0 (2.9–8.6)	<0.01
Baseline ScvO_2_, %	70 (63–77)	72 (65–78)	69 (62–77)	0.03
Six-hour ScvO_2_, %	74 (67–79)	75 (69–79)	70 (62–76)	<0.01
WBC, ×10^3^ per mm^3^	7.4 (2.1–16.5)	7.7 (2.4–16.0)	6.5 (0.7–20.0)	0.23
Hemoglobin, g/dL	11.0 (9.3–12.6)	11.2 (9.7–13.0)	9.5 (8.4–11.1)	<0.01
Platelets, ×10^3^ per mm^3^	124 (61–199)	127 (70–197)	103 (34–206)	0.37
Creatinine, mg/dL	1.3 (0.9–2.0)	1.3 (0.9–1.8)	1.7 (1.1–2.4)	0.01
Total bilirubin, mg/dL	1.3 (0.9–2.3)	1.3 (0.9–2.3)	1.4 (0.8–3.2)	0.71
PT, INR	1.3 (1.1–1.5)	1.2 (1.1–1.4)	1.4 (1.2–2.1)	<0.01
Positive blood culture	138 (38)	114 (38)	24 (37)	0.84
SOFA score	8 (6–10)	7 (5–9)	10 (9–12)	<0.01
APACHE II score	17 (13–22)	16 (13–21)	23 (18–30)	<0.01
Sepsis intervention				
Total fluids, 0–6 h, L	3.5 (2.6–4.4)	3.5 (3.0–4.2)	3.5 (2.5–4.4)	0.41
Use of vasopressors within 6 h	301 (83)	243 (82)	58 (89)	0.14
Use of dobutamine within 6 h	51 (14)	39 (13)	12 (18)	0.26
RBC transfusion within 6 h	66 (18)	48 (16)	18 (28)	0.03
Mechanical ventilation	51 (14)	28 (9)	23 (35)	<0.01
Resuscitation bundle				
Timely antibiotic use*	238 (66)	195 (65)	43 (66)	0.91
Fluid challenge (20ml/kg)	349 (96)	292 (98)	57 (88)	<0.01
CVP ≥8 mmHg achieved	287 (97)	238 (80)	49 (75)	0.42
MAP ≥65 mmHg achieved	359 (99)	295 (99)	64 (98)	0.71

Data are presented as the median (interquartile range) or number (%).

*Timely antibiotic use: broad spectrum antibiotics administered within three hours from the time of presentation if the patient was hypotensive and/or had a lactate level ≥ 4 mmol/L

MAP, mean arterial pressure; ScvO_2_, central venous oxygen saturation; WBC, white blood cell count; PT, prothrombin time; INR, international normalized ratio; SOFA, sequential organ failure assessment; APACHE II, acute physiology and chronic health evaluation II; RBC, red blood cells: CVP, central venous pressure.

**Fig 1:**
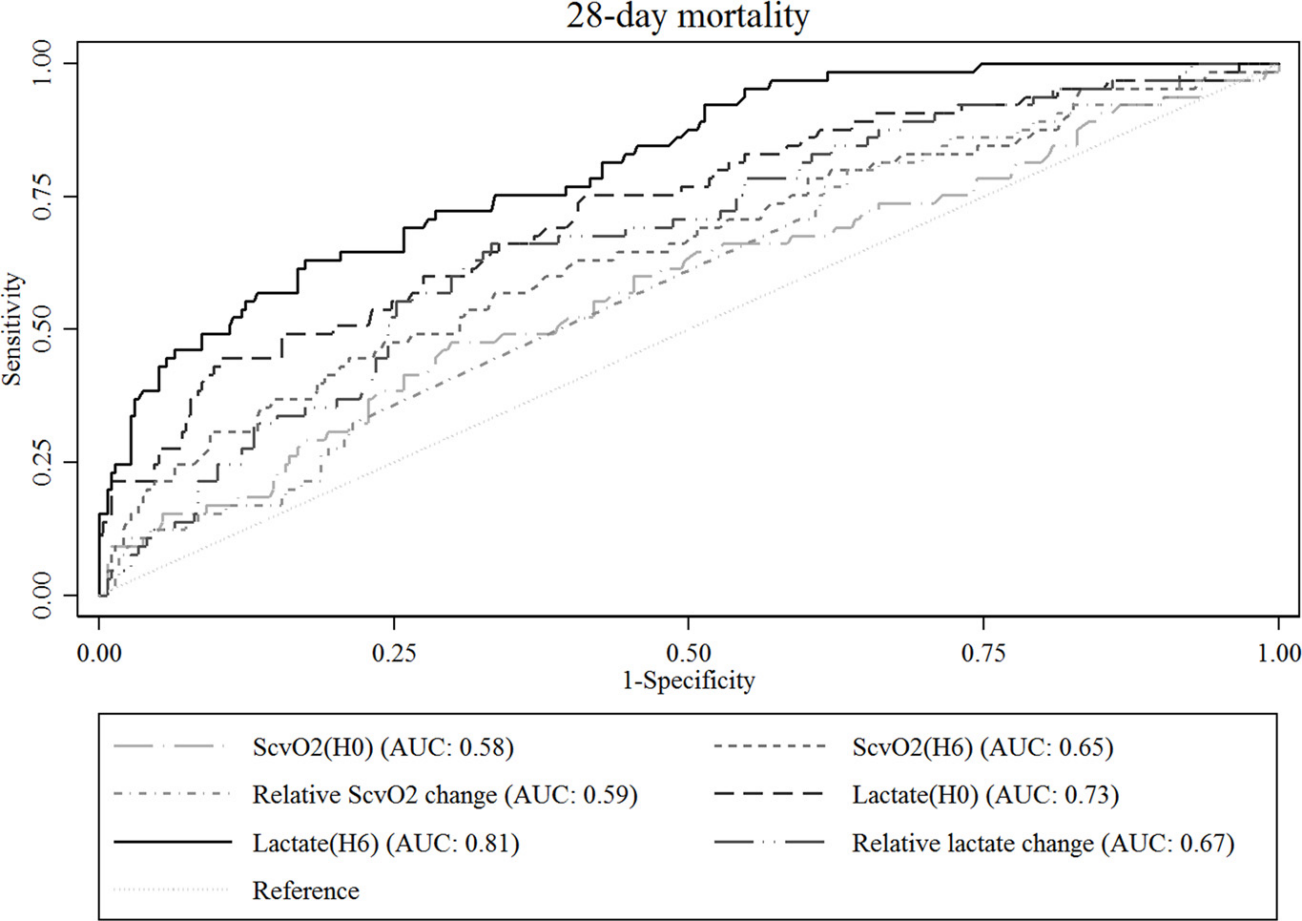
Receiver operating characteristic curves of ScvO_2_ and serum lactate for predicting 28-day mortality. ScvO_2_ (H0) and lactate (H0), initial ScvO_2_ and serum lactate at the time of presentation with severe sepsis or septic shock, respectively; ScvO_2_ (H6) and lactate (H6), ScvO_2_ and serum lactate 6 hours after presentation, respectively; Relative ScvO_2_ change, [(ScvO_2_(H0)–ScvO_2_(H6))/ ScvO_2_(H0)] x 100 (%); relative lactate change, [(lactate(H0)–lactate(H6))/ lactate(H0)] x 100 (%).

ROC curve analysis showed that lactate (H6) had the highest AUC for predicting 28-day mortality, which was 0.81 (95% confidence interval [CI], 0.75–0.87) (Fig 1). AUC values for the other variables were as follows: lactate (H0), 0.73 (95% CI, 0.66–0.80); relative lactate change, 0.67 (95% CI, 0.60–0.74); ScvO_2_ (H6), 0.65 (95% CI, 0.57–0.73); relative ScvO_2_ change, 0.59 (95% CI, 0.51–0.66); ScvO_2_ (H0), 0.58 (95% CI, 0.50–0.66). There were significant differences between lactate (H6) and other variables in the ROC curves (*P* <0.01).

According to lactate normalization and ScvO_2_ (H6), 106 (29%) were classified into Group 1, 40 (11%) into Group 2, 137 (38%) into Group 3, and 80 (22%) into Group 4 (Table 2). Baseline characteristics of each group are summarized in Table 2. Age, sex, comorbidities (except hypertension and liver cirrhosis), and initial hemodynamics were not significantly different among the four groups. However, the levels of initial serum lactate and ScvO2 (H0) were significantly different among the groups. In addition, the initial SOFA score and APACHE II score were also significantly different among the groups.

**Table 2:** Comparison of baseline characteristics of the subgroups.

	Lactate normalization		No lactate normalization		*P* value
	ScvO_2_ (H6) ≥70 (Group 1, n = 106)	ScvO_2_ (H6) <70 (Group 2, n = 40)	ScvO_2_ (H6) ≥70 (Group 3, n = 137)	ScvO_2_ (H6) <70 (Group 4, n = 80)	
Age, years	65 (50–72)	67 (60–73)	63 (54–71)	67 (56.5–73)	0.23
Sex, male	61 (57.6)	20 (50.0)	72 (52.6)	51 (63.8)	0.35
Comorbidities					
Hypertension	35 (33.0)	18 (45.0)	35 (25.6)	34 (42.5)	0.03
Diabetes	21 (19.8)	5 (12.5)	36 (26.3)	12 (15.0)	0.12
Chronic lung disease	6 (5.7)	3 (7.5)	7 (5.1)	4 (5.0)	0.94
Cardiovascular disease	8 (7.6)	2 (5.0)	8 (5.8)	14 (17.5)	0.02
Chronic renal failure	6 (5.7)	0 (0.0)	2 (1.5)	5 (6.3)	0.10
Liver cirrhosis	4 (3.8)	0 (0.0)	18 (13.1)	4 (5.0)	0.01
Malignancy	58 (54.7)	26 (65.0)	73 (53.3)	48 (60.0)	0.56
Site of infection					
Intra-abdominal	30 (28.3)	12 (30.0)	32 (23.4)	37 (46.3)	0.01
Respiratory tract	46 (24.6)	35 (38.0)	16 (28.6)	14 (50.0)	0.01
Urinary tract	19 (17.9)	4 (10.0)	24 (17.5)	6 (7.5)	0.12
Other	18 (17.0)	11 (27.5)	24 (17.5)	13 (16.3)	0.44
Initial vital signs					
Initial MAP	62 (55–67)	63 (59–73.5)	65 (56–77)	64 (56–73)	0.15
Heart rate	113 (95–129)	119 (98.5–135)	111 (94–127)	118 (101.5–140.5)	0.07
Respiratory rate	20 (20–22)	20 (19.5–22)	20 (20–24)	23 (20–28)	0.02
Temperature	38.2 (37.1–38.8)	37.8 (36.8–38.9)	38.1 (36.8–39.0)	37.9 (36.8–38.9)	0.49
Laboratory tests					
Baseline lactate, mmol/L	2.3 (1.5–3.6)	2.7 (1.4–4.6)	5.4 (4.0–6.8)	4.8 (3.5–6.9)	<0.01
Six-hour lactate, mmol/L	1.3 (1.0–1.6)	1.4 (1.1–1.7)	3.8 (2.7–5.6)	3.9 (2.6–5.6)	<0.01
Baseline ScvO_2_, %	72 (67–78)	65 (58–69)	72 (66–78)	63 (57–72)	<0.01
Six-hour ScvO_2_, %	76 (74–80)	65 (62–67)	77 (74–81)	64 (57–67)	<0.01
WBC, ×10^3^ per mm^3^	10.3 (4.4–17.8)	7.2 (0.9–17.8)	6.3 (2.0–15.6)	4.6 (0.8–14.0)	0.04
Haemoglobin, g/dL	11.1 (9.7–12.9)	11.0 (9 .0–12.6)	11.2 (9.4–13.0)	10.3 (8.7–12.2)	0.30
Platelets, ×10^3^ per mm^3^	156 (85–217)	132 (62–233)	104 (42–178)	112 (52–194)	0.01
Creatinine, mg/dL	1.09 (0.78–1.82)	1.20 (0.85–1.85)	1.43 (1.02–1.93)	1.45 (1.00–2.09)	<0.01
Total bilirubin, mg/dL	1.2 (0.7–1.9)	1.3 (0.9–2.1)	1.7 (1.1–3.5)	1.2 (0.8–1.9)	<0.01
PT, INR	1.17 (1.06–1.30)	1.23 (1.09–1.43)	1.32 (1.16–1.60)	1.31 (1.16–1.57)	<0.01
Positive blood culture	27 (25.5)	15 (37.5)	71 (51.8)	25 (31.3)	<0.01
SOFA score	7 (5–8)	7.5 (5–9.5)	9 (6–10)	9 (6–11)	<0.01
APACHE II score	15.5 (12–19)	18.5 (13–23)	17 (13–22)	20 (16–22.5)	<0.01

Data are presented as median (interquartile change) or number (%).

ScvO_2_, central venous oxygen saturation; MAP, mean arterial pressure; PT, prothrombin time; INR, international normalised ratio; SOFA, sequential organ failure assessment; APACHE II, acute physiology and chronic health evaluation II.

**Fig 2:**
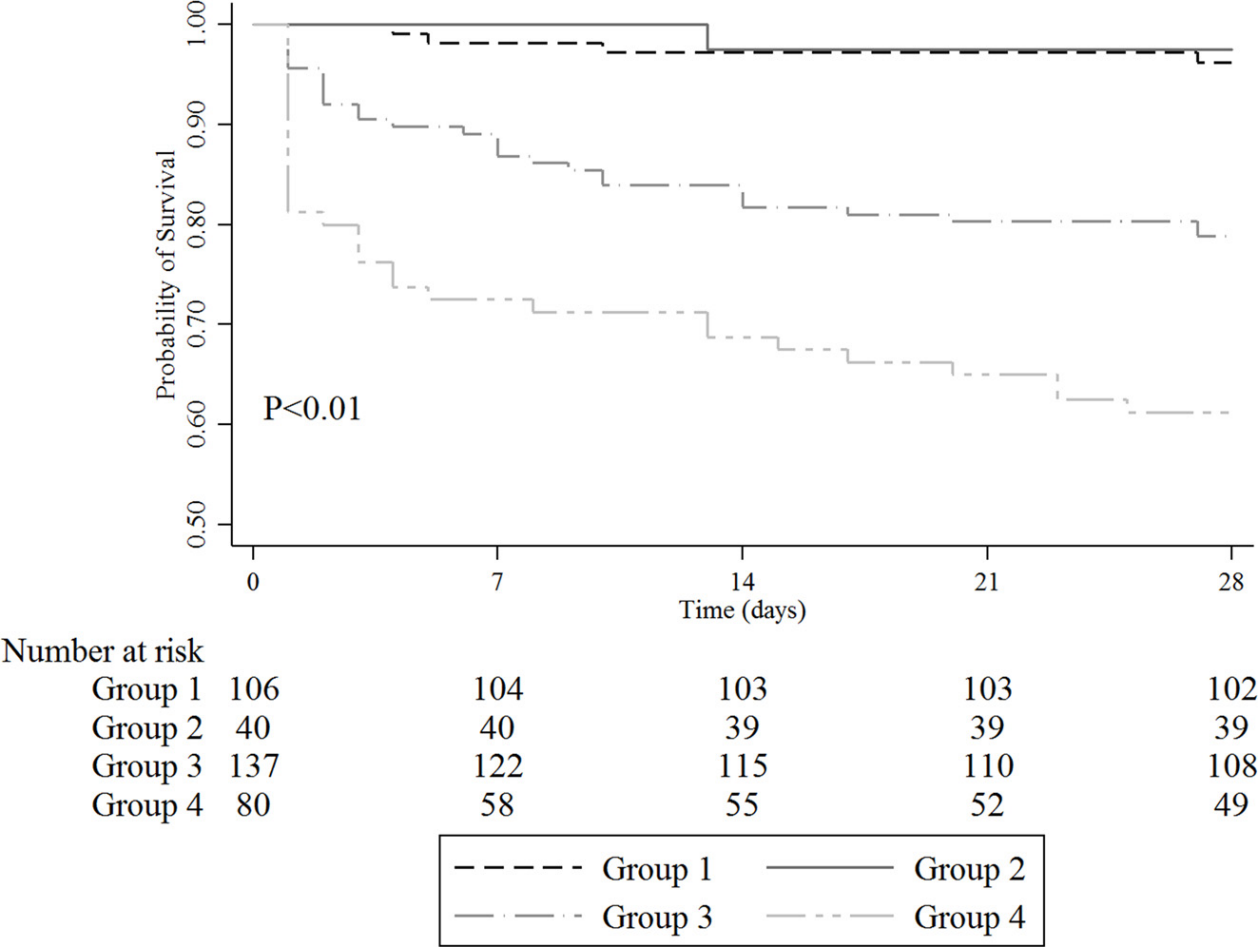
Kaplan-Meier survival analysis plot for 28-day mortality. Group 1, Patients with lactate normalization and ScvO_2_ ≥70%; Group 2, patients with lactate normalization and ScvO_2_ <70%; Group 3, patients without lactate normalization and ScvO_2_ ≥70%; Group 4, patients without lactate normalization and ScvO_2_ <70%.

A comparison of outcomes is shown in Table 3. There were significant differences in 28-day mortality among the four groups. The 28-day mortality of Group 4 (39%) was the highest followed by that of Group 3 (22%). Patients with lactate normalization showed significantly lower 28-day mortality compared to patients without lactate normalization (3% vs. 28%, *P* <0.01). However, for the achievement of ScvO_2_ (H6) ≥70%, there was a significant difference in 28-mortality only in patients without lactate normalization (21% vs. 39%, *P <*0.01), while no difference was observed in patients with lactate normalization (4% vs. 3%, *P* = 0.71). The Kaplan-Meier curves showed similar results (Fig 2). There were significant differences between the four groups in subgroup analysis (*P* <0.01 by log-rank test), except between Groups 1 and 2 (*P* = 0.70 by log-rank test).

**Table 3:** Comparison of outcomes.

Outcomes	Total (N = 363)	Lactate normalization		No lactate normalization		*P* value
		ScvO_2_ (H6) ≥70 (Group 1, n = 106)	ScvO_2_ (H6) <70 (Group 2, n = 40)	ScvO_2_ (H6) ≥70 (Group 3, n = 137)	ScvO_2_ (H6) <70 (Group 4, n = 80)	
28-day mortality	65 (18)	4 (4)	1 (3)	29 (21) ^*^^†^	31 (39) ^*^^†^^‡^	<0.01
In-hospital mortality	70 (19)	8 (8)	3 (8)	33 (24) ^*^	26 (33) ^*^^†^	<0.01
In-hospital LOS^§^, days	12 (8–22)	11 (8–20)	12 (7–20)	14 (9–24.5)	11.5 (8–21)	0.13

Data are presented as the median (interquartile change) or number (%).

*P < 0.05 compared to Group 1 after Bonferroni correction.

†*P* < 0.05 compared to Group 2 after Bonferroni correction.

‡*P* < 0.05 compared to Group 3 after Bonferroni correction.

LOS, length of stay.

§ Only survivors were included in analysis.

In addition, 28-day mortality, according to ScvO_2_ (H6) quartiles was 31% in the first quartile (41–67%), 15% in the second quartile (68–74%), 14% in the third quartile (75–79%), and 12% in the fourth quartile (79–92%) (*P* <0.01). No significant differences were observed in the subgroup with lactate normalization.

The results of univariable and multivariable analysis for 28-mortality are shown in Table 4. In multivariable logistic regression analysis, the adjusted OR for 28-day mortality was 0.41 (95% CI, 0.04–3.99, *P* = 0.44) for Group 2, 2.62 (95% CI, 0.79–8.77, *P* = 0.12) for Group 3, and 6.36 (95% CI 1.92–21.01, *P* < 0.01) for Group 4 when compared to Group 1.

According to another logistic model using ScvO_2_ (H6) and lactate normalization instead of subgroup variables, lactate normalization was significantly associated with 28-day mortality (adjusted OR for 28-day mortality, 0.20; 95% CI, 0.07–0.54; *P* <0.01), while ScvO_2_ (H6) ≥70% showed only a marginal association (adjusted OR for 28-day mortality, 0.51; 95% CI, 0.26–1.01; *P* = 0.05). ScvO_2_ (H6) ≥70% was associated with 28-day mortality only in cases without lactate normalization on additional subgroup analysis (adjusted OR 0.37, 95% CI, 0.18–0.79; *P* = 0.01).

**Table 4:** Univariable and multivariable analysis for 28-day mortality.

Variable	Univariable		Multivariable	
	Unadjusted OR (95% CI)	*P* value	Adjusted OR (95% CI)	*P* value
Subgroups				
Group 1	Reference		Reference	
Group 2	0.65 (0.07–6.03)	0.71	0.41 (0.04–3.99)	0.44
Group 3	6.85 (2.33–20.16)	<0.01	2.62 (0.79–8.77)	0.12
Group 4	16.13 (5.39–48.25)	<0.01	6.36 (1.92–21.01)	<0.01
Age, years	1.01 (0.99–1.03)	0.23	1.02 (0.99–1.04)	0.20
Gender, male	0.89 (0.52–1.53)	0.67	0.53 (0.26–1.09)	0.08
Comorbidities				
Hypertension	1.19 (0.68–2.09)	0.53		
Diabetes	0.86 (0.43–1.71)	0.67		
Chronic lung disease	0.49 (0.11–2.18)	0.35		
Cardiovascular disease	1.60 (0.69–3.75)	0.28		
Chronic renal failure	1.39 (0.37–5.21)	0.62		
Liver cirrhosis	1.10 (0.40–3.03)	0.86		
Malignancy	1.51 (0.87–2.64)	0.15		
Site of infection				
Intra-abdomen	Reference		Reference	
Respiratory tract	2.91 (1.47–5.76)	<0.01	3.20 (1.39–7.35)	<0.01
Urinary tract	0.64 (0.20–2.03)	0.45	0.47 (0.11–2.04)	0.31
Other	2.52 (1.16–5.48)	0.02	2.81 (1.07–7.37)	0.04
Baseline lactate (+1 mmol/L)	1.32 (1.20–1.44)	<0.01	1.22 (1.10–1.36)	<0.01
Baseline ScvO_2_ ≥70%	0.61 (0.35–1.04)	0.07	0.94 (0.46–1.89)	0.86
SOFA score (+1)	1.27 (1.16–1.39)	<0.01	1.17 (1.05–1.30)	<0.01
Resuscitation bundle				
Timely antibiotic use	0.49 (0.25–0.95)	0.04	0.38 (0.16–0.88)	0.02
Intravenous fluid challenge	0.15 (0.05–0.44)	<0.01	0.56 (0.14–2.16)	0.40
CVP ≥8 mmHg achieved	0.77 (0.41–1.45)	0.42		
MAP ≥65 mmHg achieved	0.65 (0.67–6.36)	0.71		

OR, odds ratio; CI, confidence interval; ScvO_2_, central venous oxygen saturation; SOFA, sequential organ failure assessment; timely antibiotic use, broad spectrum antibiotics administered within three hours from the time of presentation if the patient was hypotensive and/or had a lactate level ≥ 4 mmol/L; CVP, central venous pressure; MAP, mean arterial pressure.

## Discussion

In the present study, we found that lactate level 6 hours after resuscitation was the most significant predictor of 28-day mortality compared with initial lactate, relative lactate change, initial ScvO_2,_ 6-hour ScvO_2,_ and relative ScvO_2_ change. Our study revealed that ScvO_2_ has some limitations as a predictor for outcome and that ScvO_2_ has no further prognostic value under lactate normalisation after initial resuscitation. These findings might be relevant in reference to recent clinical trials indicating that strict protocolized resuscitation including optimisation of ScvO_2_ ≥70% does not lead to better survival and suggesting that not all patients with septic shock require central venous access and invasive monitoring [4–6]. Our study also revealed that ScvO_2_ might be predictive in patients without lactate normalisation, suggesting the need for further therapeutic intervention. The finding suggests that ScvO_2_ can be selectively measured during resuscitation to obtain more clinical information in refractory cases.

Lactate and ScvO_2_ can be used as targets of initial resuscitation in patients with severe sepsis or septic shock [7]. However, there is a discrepancy between lactate and ScvO_2_ regarding their correlation with mortality [8, 26]. Therefore, these values should be interpreted in the clinical context, in a complementary sense. Theoretically, patients could be categorised into four oxygenation groups that might reflect underlying pathophysiologic conditions: group 1, relatively normal or compensated metabolic status; group 2, oxygen deficit status; group 3, microcirculatory dysfunction status; group 4, oxygen debt status [12, 15, 27]. For example, patients with high ScvO_2_ without lactate normalisation (group 3) might be in a refractory status involving impaired oxygen extraction due to microcirculatory failure or cellular dysfunction [15, 27] and appropriate therapeutic options might vary. Further research with a larger population is needed to investigate this issue.

Static lactate is often nonspecific as a single measurement and normal levels do not ensure favorable outcomes in patients with sepsis [15]. LC as a dynamic value may be a good indicator of the effectiveness of resuscitation and, moreover, is readily available and can be measured peripherally [15, 28]. Nguyen et al. [10] demonstrated that a relative LC of 10% within 6 hours was the optimal value for predicting survival after adjusting for initial lactate level. Puskarich et al. [29] proposed early lactate normalization during the first 6 hours of resuscitation as the strongest independent predictor of survival. Because lactate kinetics is too complex to interpret accurately in a clinical setting, a reasonable approach might be repeated measurement in order to monitor trends in lactate levels and target normalisation as an ultimate goal, as the current international guidelines recommend [1]. The results of our study, that 6-hour lactate was the strongest predictor of 28-day mortality, are consistent with these previous findings [10, 29].

To fully appreciate our results, the limitations of this study must be acknowledged. First, a major limitation is the fact that lactate and ScvO_2_ were not simultaneously measured in all patients eligible for the study. Fully half of the patients who met the inclusion criteria were excluded from final analysis because of this even though the data were retrieved from a prospectively collected sepsis registry. The mortality rate of the included patients was higher than that of the excluded patients, indicating that patients with more severe disease status might be included. Therefore, there is a possibility that selection bias might have influenced our findings. Furthermore, our study population was from a single institution with an early quantitative resuscitation program for sepsis patients who present to the ED. Thus, our findings may not be broadly applicable to other centres at which resuscitation programs are not available for sepsis management. Second, ScvO_2_ was one of targets to be achieved during initial resuscitation, while 6-hour lactate or relative lactate change would be the end results of the resuscitation. However, therapeutic interventions including aggressive fluid resuscitation, antibiotics, vasopressors and respiratory support might not be profoundly different. Third, due to the small sample size, there may have been insufficient statistical power to identify some significant findings. For example, no comorbidities were associated with mortality in our cohort, although comorbidities might be a predictor or contributing factor to mortality in sepsis patients. It could be also attributed to a stronger association of severity of organ failure by sepsis, more than comorbidity per se.

## Conclusions

Among ScvO_2_ and lactate-related variables, lactate concentration 6 hours after initial resuscitation in patients with severe sepsis or septic shock was the strongest predictor of 28-day mortality. Six-hour ScvO_2_ had no prognostic value in cases with lactate normalization, but had additional prognostic value in cases where serum lactate values were not normalized.
