# Correction: Prognostic Value of Lactate and Central Venous Oxygen Saturation after Early Resuscitation in Sepsis Patients

**DOI:** 10.1371/journal.pone.0157225

**Published:** 2016-06-03

**Authors:** Young Kun Lee, Sung Yeon Hwang, Tae Gun Shin, Ik Joon Jo, Gee Young Suh, Kyeongman Jeon

There is an error in reference 17. The correct reference is: Joo YM, Chae MK, Hwang SY, Jin S-C, Lee TR, Cha WC, et al. Impact of timely antibiotic administration on outcomes in patients with severe sepsis and septic shock in the emergency department. Clin Exp Emerg Med. 2014;1(1):35–40.
